# Emerging roles of endoplasmic reticulum stress in the cellular plasticity of cancer cells

**DOI:** 10.3389/fonc.2023.1110881

**Published:** 2023-02-20

**Authors:** Hao Wang, Kun Mi

**Affiliations:** ^1^ Breast Surgery, Sichuan Cancer Hospital & Institute, Sichuan Cancer Center, School of Medicine, University of Electronic Science and Technology of China, Chengdu, China; ^2^ Radiation Oncology Key Laboratory of Sichuan Province, Sichuan Cancer Hospital & Institute, Sichuan Cancer Center, School of Medicine, University of Electronic Science and Technology of China, Chengdu, China

**Keywords:** cellular plasticity, ER stress, epithelial-mesenchymal plasticity, resistance, cancer stem cell, vasculogenic mimicry

## Abstract

Cellular plasticity is a well-known dynamic feature of tumor cells that endows tumors with heterogeneity and therapeutic resistance and alters their invasion–metastasis progression, stemness, and drug sensitivity, thereby posing a major challenge to cancer therapy. It is becoming increasingly clear that endoplasmic reticulum (ER) stress is a hallmark of cancer. The dysregulated expression of ER stress sensors and the activation of downstream signaling pathways play a role in the regulation of tumor progression and cellular response to various challenges. Moreover, mounting evidence implicates ER stress in the regulation of cancer cell plasticity, including epithelial–mesenchymal plasticity, drug resistance phenotype, cancer stem cell phenotype, and vasculogenic mimicry phenotype plasticity. ER stress influences several malignant characteristics of tumor cells, including epithelial-to-mesenchymal transition (EMT), stem cell maintenance, angiogenic function, and tumor cell sensitivity to targeted therapy. The emerging links between ER stress and cancer cell plasticity that are implicated in tumor progression and chemoresistance are discussed in this review, which may aid in formulating strategies to target ER stress and cancer cell plasticity in anticancer treatments.

## 1 Introduction

Cancer cell plasticity refers to the dynamic transition of cellular state that occurs during cancer initiation and progression (1, 2), which contributes to tumor heterogeneity and therapeutic resistance (3, 4). Epithelial-to-mesenchymal transition (EMT) and, the reversed process, mesenchymal-to-epithelial transition (MET) are the well-known forms of cellular plasticity, representing fundamental processes in the tumor invasion–metastasis cascade (5, 6). Epithelial–mesenchymal plasticity (EMP) encompasses EMT and MET, which are the key phenomena in tumor metastasis that are associated with cancer stem cell (CSC) generation and maintenance and therapeutic resistance (7–9), thereby posing a major challenge to effective therapy. Furthermore, CSCs exhibit epigenetic plasticity and therapeutic resistance, which contribute to cancer progression or relapse. Recent evidence also suggests that drug-resistant cells possess abnormal energetic and metabolic pathways that are involved in the induction, maintenance, and alteration of multidrug resistance (MDR) phenotype (10, 11).

The ER is a central organelle that facilitates protein synthesis, assembly, folding, and modification. The retention of unfolded or misfolded proteins within the ER lumen triggers the unfolded protein response (UPR), which leads to ER stress. Because of its roles in the regulation of multiple cancer cell functions, increasing evidence has linked ER stress to tumor progression (12, 13). ER stress has been shown to influence cancer cell proliferation, apoptosis, inflammatory response, and metastatic capacity (14). It has also been widely observed that when exposed to physiologic or pathologic stresses, cancer cells adopt various identities along a phenotypic spectrum, which results in cellular plasticity. However, the links between ER stress and cancer cell plasticity, such as EMP, MDR phenotype, CSC phenotype, and vasculogenic mimicry (VM) phenotype plasticity, have not been completely investigated, and new evidence is emerging. Here, we reviewed the roles of ER stress in cancer cell plasticity and the underlying molecular mechanisms.

## 2 Cellular plasticity in cancer

**Figure 1:**
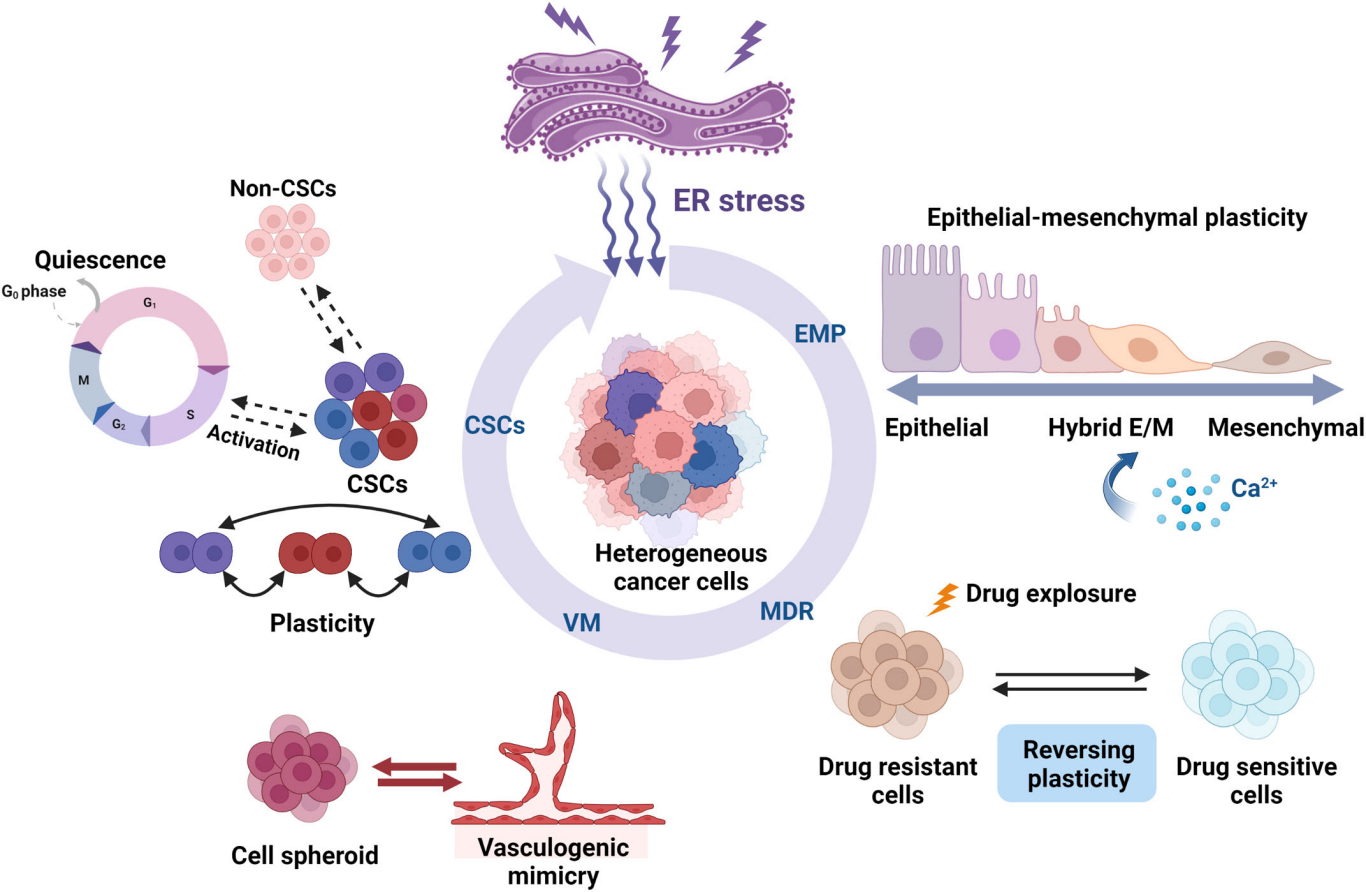
Roles of ER stress in the cellular plasticity of cancer cells. ER stress regulates cancer cell plasticity, including EMP, drug resistance phenotype, CSC phenotype, and VM phenotype plasticity. ER stress can regulate the EMP of cells that migrate along the EMT–MET axis, such as activation, inhibition, and pEMT induction. Several studies have found that ER stress influences the cell fate by promoting or suppressing the susceptibility to drug therapy. ER stress also plays an important role in regulating CSC differentiation and ratio and also regulates CSC plasticity, such as quiescence and activation. The interchange and coexistence of diverse phenotypes need to be studied further. VM is also another remarkable example of tumor cell plasticity. The activation of ER stress inhibits the formation of VM phenotypes in highly aggressive cells, thereby impeding tumor angiogenesis and progression. Created with BioRender.com.

Cellular plasticity, which is observed during development, injury, and tumor progression, is a critical process that allows cells to assume distinct phenotypes to adapt to changing conditions (1, 15). Cellular plasticity is important in tumor proliferation, invasion, metastasis, and chemoresistance (16). Tumor cells can undergo phenotypic switch in response to cues from the surrounding microenvironment, such as EMP, CSC plasticity, drug resistance plasticity, and transdifferentiation, including VM (**Figure 1**).

### 2.1 EMP

Various human cancers exhibit plasticity between epithelial and mesenchymal states and the presence of EMT, MET, and hybrid epithelial/mesenchymal (E/M) or partial EMT (pEMT) phenotypes (17–20). EMT is defined as epithelial cells gradually losing epithelial characteristics while gaining motility and invasive characteristics of mesenchymal cells. MET is the reverse of EMT in that the cellular phenotype changes from mesenchymal cells to epithelial cells, thereby regaining apical–basal polarity. EMP refers to the ability of tumor cells to differentiate along the epithelial-mesenchymal spectrum and exhibit various intermediate hybrid E/M states (21, 22). As evidenced by pEMT, cells shift along the EMT-MET axis, which implies that EMT and MET are not binary fates (6). The underlying topographic map of EMT reveals a plethora of metastable hybrid phenotypic states, thus distinguishing stable epithelial and mesenchymal states (23). EMT, MET, and pEMT states can differ depending on tumor types, dissemination states, and the degrees of metastatic colonization or dormancy (7). In triple-negative breast cancer (TNBC), for example, EMP is a crucial mechanism that contributes to phenotype plasticity and heterogeneity, resulting in a heterogeneous clinical behavior associated with a poor prognosis (24). Primary breast CSCs of TNBC express epithelial and mesenchymal markers, thus indicating an EMP state (25). EMP is found in the majority of heterogeneous circulating tumor cell(CTC) phenotypes in the CTCs of patients with breast cancer (26).

In addition to the well-known transcriptional and post-transcriptional regulation that underpins EMP (27), plasticity is epigenetically regulated. Changing specific chromatin modifications demonstrates the roles of epigenetic regulators during EMP. In prostate cancer, for example, suppressing the epigenetic regulator chromatin remodeling proteinHMGA2 with a histone deacetylase (HDAC) inhibitor inhibits EMP and significantly reduces tumor growth and metastasis (28). HDAC, Methyl-CpG-binding domain 3 (Mbd3)/nucleosome remodeling and deacetylase (NuRD)complex, and Ten-eleven translocation 2 (Tet2) hydroxylase have all been identified as important regulators of EMP and metastasis in breast cancer (29).

Many functional proteins are involved in EMP regulation. The coxsackie and adenovirus receptor (CXADR), a tight junction protein, stabilizes AKT regulators and controls EMP in breast cancer (30). Syndecan-1, a cell surface proteoglycan, regulates EMP in tumor cells *via* nuclear translocation (31). Snail activation mediates EMP induction in prostate cancer (32). EMP regulators may also accommodate dynamic changes. The expression of the cell adhesion molecule CD44 is complex, with many isoforms, and the pattern of isoform expression changes during EMP transitions (33). Dynamic changes in the cohesin subunit RAD21 mediate chromatin architecture to control EMP for the regulation of cell fate in breast and gastric cancers (34). Furthermore, certain proteins can mediate the EMP process in a two-way manner. Secretary osteopontin (OPN) activates EMT to initiate cancer metastasis, whereas intracellular OPN (iOPN) induces MET to promote metastasis (35). Similarly, the kinesine-1 subunits kinesin family member 5B (KIF5B)/kinesin light chain 1 (KLC1) modulate the EMP process differently in breast cancer, with KIF5B being an inducer of EMT and KLC1 being its suppressor (36). Exosomes (37) and the classical transforming growth factor (TGF)β signaling pathway can also regulate tumor cell EMP (38, 39). Furthermore, the tumor microenvironment influences EMP regulation. Cancer-associated fibroblasts, for example, drive EMP and the formation of hybrid E/M states to induce invasive and metastatic tumor cell clusters (40). The EMP process also involves various phenotypic subtypes of macrophages in the tumor microenvironment (41).

EMP is a key mediator of metastatic dissemination and therapeutic resistance in several solid tumors. By restraining the EMP of tumor cells, researchers may be able to inhibit the progression of metastasis by facilitating an asymptomatic state of dormancy. A recent study has revealed that inhibition of spleen tyrosine kinase increases systemic tumor dormancy and, thus, reduces breast cancer metastasis (42).

### 2.2 Plasticity in drug resistance

According to emerging evidence, cancer therapies are hampered by reversible mechanisms that cause drug resistance. The plasticity of cancer cells drives their transformation to a phenotypic state that is not dependent on the original drug-responsive pathway. Because of intratumor heterogeneity and ongoing diversification in response to therapy, tumor cells survive the treatment and develop new resistant phenotypes (43). In melanoma, phenotype plasticity is a major cause for therapeutic resistance and is associated with increased levels of epidermal growth factor receptor (EGFR), receptor tyrosine kinaseAXL, or nerve growth factor receptor (NGFR), the expression of which is further upregulated by v-raf murine sarcoma viral oncogene homolog B1 (BRAF) inhibitors (44). Hence, therapeutic strategies could capitalize on this reversibility before relapse tumors develop genetic alterations that drive resistance. Furthermore, plasticity allows tumor cells to switch reversibly between drug resistance and drug sensitivity to escape and survive therapeutic challenges (45).

According to new research, a drug-tolerant population can switch between drug-sensitive and drug-tolerant states *via* non-genetic mechanisms, such as chromatin remodeling, and evolve into various resistant phenotypes (46). Plasticity in chemotherapy resistance is reflected in non-small cell lung cancer (NSCLC) by epigenetic alterations that allow tumor cells to adapt to new microenvironments after drug administration (47). Tamoxifen treatment causes acquired drug resistance in MCF7 breast cancer cells by altering the DNA methylation status (48). It has been demonstrated that epigenetic silencing of Spalt-like transcription factor 2 (SALL2) contributes to tamoxifen resistance in breast cancer by activating the AKT/mTOR pathway (49). Moreover, the ETS1/miR-23a-3p/ACSL4 axis may mediate sorafenib resistance *via* epigenetic regulation of ferroptosis in human hepatocellular carcinoma (50). Recent research provides more evidence demonstrating the crucial roles of epigenetic changes in regulating the resistant phenotype of tumor cells, which could serve as promising targets for overcoming clinical resistance. Furthermore, by activating cellular plasticity, tumor cells may be able to create a tumor-supportive microenvironment. The highly plastic cells in this microenvironment change dramatically to resist therapeutic drugs (51).

### 2.3 CSC plasticity

CSCs are a subpopulation of tumor cells capable of self-renewal and tumorigenesis. CSCs retain high stemness and plasticity (52), as evidenced by the processes of non-CSCs becoming CSCs, CSCs losing stemness, quiescent CSCs becoming active, and CSCs becoming quiescent. Non-CSCs in human basal breast cancers, for example, can acquire CSC phenotypes when zinc finger e-box binding homeobox 1 (ZEB1) is activated (53). Intravital lineage tracing in mammary tumors shows that existing CSCs disappear and new CSCs are formed during mammary tumor growth, thereby demonstrating the dynamic nature of plasticity in these cells (54). Cells expressing CSC-associated markers in glioblastoma do not represent a clonal entity but rather a plastic state that most cancer cells can adapt in response to microenvironmental signals (55). The dynamic coexistence of various phenotypes or states in CSCs is becoming common in various tumor contexts. Malignant cells in glioblastoma exist in four major cellular states that can interconvert and exhibit plasticity, which drives intratumoral heterogeneity (56). CSCs have been shown to exhibit three interchangeable phenotypes in breast cancer, namely, ALDH^+^, CD44^+^CD24^−^, and ALDH^+^CD44^+^CD24^−^ CSCs, which indicates the plasticity and heterogeneity of CSCs (57).

CSCs can enter a dormant cellular state and exist in the G_0_ phase, which makes them resistant to conventional therapies that target actively dividing cells. Quiescence can be induced by altered microenvironmental cues or drug treatments. Breast cancer disseminated tumor cells (BC DTCs) may be instructed to enter dormancy by bone marrow NG2^+^/Nestin^+^ mesenchymal stem cells. When homeostasis of the bone marrow microenvironment changes, BC DTCs may emerge and cause a bone relapse (58). Laminin-332, as a component of the human hepatic CSC niche, plays a role in sustaining cell stemness and confers chemoresistance and quiescence (59). One understudied chemoresistance mechanism is the induction of quiescence. Nuclear factor of activated T cells cytoplasmic 4 (NFATC4) drives a quiescent phenotype in ovarian cancer and promotes chemotherapy resistance *in vitro* and *in vivo (* 60). Apart from resistance to therapy, epigenetic determinants play an important role in CSC dormancy (61). SET domain-containing protein 4 (SETD4) alters heterochromatin formation to epigenetically regulate CSC quiescence in breast cancer (62). Alterations in cell states and switches to a dormant or quiescent state are major impediments to standard therapy (63).

Other studies indicate that quiescent CSCs can be reactivated under favorable conditions (64, 65). Transfer of mitochondrial DNA from extracellular vesicles acts as an oncogenic signal, potentially promoting the emergence of dormant cancer stem-like cells (66). Nuclear protein DEK is required for CSC activation in breast tumors as it upregulates cellular activation-related genes, including MYC targets (67). Understanding the mechanism underlying the activation of quiescent CSCs may lead to novel therapeutic strategies for overcoming quiescence-linked chemoradiotherapy resistance.

### 2.4 Plasticity in VM

VM is another remarkable example of tumor cell plasticity. VM is a functional microcirculation structure that is independent of endothelial vessels and describes the plasticity of highly aggressive tumor cells to develop vasculogenic-like, matrix-rich networks, thus mimicking endothelial cell activities and providing blood supply for tumor growth and metastasis (68, 69). EMT, which is based on EMP, plays a crucial role in the formation of VM during cancer progression. TGF-β1/ROCK signaling contributes to the formation of VM in hepatocellular carcinoma by inducing EMT (70). In ovarian carcinoma (71) and salivary adenoid cystic carcinoma, hypoxia may promote VM formation by inducing EMT (72). Dickkopf-1 promotes VM formation in NSCLC by increasing the expression of EMT-associated proteins (73). VM formation is influenced by several known EMP regulators, including ZEB1 (74), Twist1 (75), Snail (76), and Slug (77). The discovery of mechanisms underlying VM plasticity will shed light on the search for more precise targets in antiangiogenic treatment.

## 3 ER stress and cancer cell plasticity

According to emerging evidence, ER stress appears to play an important role in regulating cellular plasticity. Chronic ER stress promotes immunosuppressive phenotypes of immune cells in various diseases, such as cancer and inflammation (78) (79). inositol-requiring enzyme 1α (IRE1α)−X-Box Binding Protein 1 (XBP1) signaling, for example, promotes tumor immune evasion by enhancing the functions of tumor-associated myeloid cells (80, 81). Furthermore, thapsigargin-induced ER stress increases interleukin(Il)-10 transcription and promotes T cell phenotype plasticity (82). Recent studies in tumor cells show a comprehensive relationship between ER stress and cancer cell plasticity (**Figure 1**), including EMP, drug resistance phenotype, CSC phenotype, and VM phenotype plasticity. In the following sections, the emerging roles of ER stress in the regulation of tumor cell plasticity and the underlying mechanisms have been discussed.

### 3.1 ER stress and EMP

**Figure 2:**
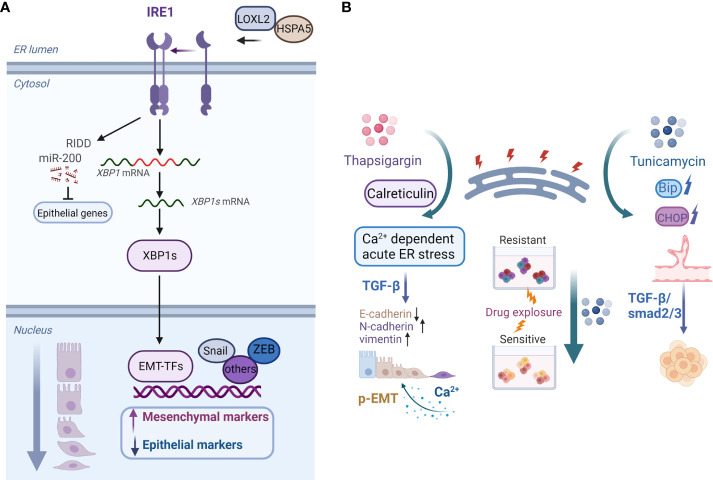
**(A)** Model of the IRE1–XBP1 axis that regulates EMP. Activation of the IRE1–XBP1 signaling pathway induces the expression of EMT transcription factors, which are direct transcriptional targets of XBP1. Additionally, IRE1α promotes miR-200 degradation *via* the RIDD process, which results in the derepression of epithelial gene transcriptional repressors. ER accumulation of LOXL2 interacts with HSPA5, activating the IRE1–XBP1 signaling pathway and inducing EMT. **(B)** Examples of roles of the ER stress inducers thapsigargin and tunicamycin in cancer cell plasticity regulation. Calreticulin promotes TGF-β-induced EMT by repressing E-cadherin and inducing N-cadherin and vimentin. Calreticulin induces EMT *via* Ca^2+^-dependent thapsigargin-induced acute ER stress. Moreover, prolonged calcium signaling induces pEMT in carcinoma cells. On the contrary, tunicamycin-induced ER stress inhibits the chemoresistance of hypopharyngeal carcinoma cells in 3D cultures. Furthermore, activation of ER stress inhibits the formation of VM phenotypes in TNBC cells *via* the TGF-β1/Smad2/3 signaling pathways.Created with BioRender.com.

In recent years, the modulatory role of ER stress in EMP in various types of tumors has been studied. Increased ER stress and EMT, for example, have been linked to chemoresistance and poor survival in patients with lung cancer. ER stress caused by the activates of valosin-containing protein disrupt the EMT-like state and promote the migratory and invasive abilities of lung cancer (83). By inducing ER stress, IL-32 promotes EMT in human lung adenocarcinoma cells (84). The downstream signaling of the ER stress sensor IRE1α acts as an EMT regulator (**Figure 2A**). IRE1α promotes lung cancer progression and EMT *via* XBP1 mRNA splicing (85). Furthermore, IRE1α promotes miR-200 degradation in an IRE1-dependent decay (RIDD)-dependent manner (86), thereby leading to the depression of epithelial gene transcriptional repressors (**Figure 2A**) **(** 87). Furthermore, sXBP1 is linked to the enhanced mesenchymal phenotypes of tumor cells. The IRE1–sXBP1 axis may be activated in response to stressful extracellular conditions that cause ER stress and regulate the expression of EMT transcription factor. The interaction of lysyl oxidase-like 2 (LOXL2) and Heat Shock Protein Family A (Hsp70) Member 5 (HSPA5) in the ER activates IRE1–XBP1 signaling and induces the expression of EMT markers in an XBP1-dependent manner (**Figure 2A**) **(** 88).

ER stress induces the expression of cyclase-associated protein 2 (CAP2) and promotes EMT *via* the activation of Rac1 and ERK in liver cancer cells (89). In hepatocellular carcinoma cells, the expression of hepatitis B virus surface antigen induces ER stress, which increases the expression and secretion of fibroblast growth factor 19 (FGF19) to activate JAK2/STAT3 signaling and induce EMT (90). Additionally, as key players of UPR, activating transcription factor 6 (ATF6) upregulation and ATF4 downregulation activates PI3K/AKT/mTOR signaling but reduces Bone Morphogenetic Protein 2 (BMP2) signaling in colorectal cancer cells to enhance motility and invasion *via* EMT (91). ER stress also promotes the overexpression of T-synthase-specific molecular chaperone Cosmc in human colorectal cancer cells, which significantly enhances cell migration and invasion *via* activation of EMT (92). In squamous cell carcinomas, ER stress triggers the ectopic expression of Transmembrane and tetratricopeptide repeat containing protein 3 (TMTC3), which activates the GRP78/Protein kinase RNA-like ER kinase (PERK) signaling and increases the expression of EMT markers *via* an interleukin-like EMT inducer (93). ER stress suppression decreases the metastatic capacities of TNBC cells by inhibiting the Syntenin/SOX4/Wnt/β-catenin pathway, whereas heat shock protein A4 overexpression reverses these effects (94). Chemotherapeutic drugs commonly used to treat patients, such as cisplatin, gemcitabine, vinorelbine, and doxorubicin, also activate ER stress, which enhances EMT and proliferative phenotypes in cancer cells (95) (96).

In noncancerous cells, ER stress has been shown to exert a direct effect on EMP. ER stress, for example, induces EMT and, thus, increases the migration of lens epithelial cells (97). ER stress inhibits cell differentiation, downregulates the expression of cadherin-1 and cadherin-16, and upregulates the expression of vimentin and SNAI1, thereby indicating the loss of epithelial features and a shift toward a mesenchymal phenotype in thyroid cells (98). Alveolar epithelial cells undergo ER stress in a hypoxic microenvironment, which is accompanied by the increased expression of mesenchymal markers both *in vivo* and *in vitro (* 99). In alveolar epithelial cells, ER stress causes a decrease in the expression of epithelial markers E-cadherin and Zonula occludens-1 and an increase in the expression of mesenchymal markers S100A4 and α-smooth muscle actin (100). EMT exerts a significant effect on pulmonary fibrosis. EMT is induced in human lung epithelial cells after treatment with the ER stress inducers tunicamycin and bleomycin *via* HDAC upregulation (101). Furthermore, advanced oxidation protein products activate ER stress in proximal tubular cells and induce EMT, as evidenced by p27 and α-SMA overexpression and E-cadherin downregulation in chronic kidney disease (102). Protein arginine methyltranferase-1 (PRMT1) causes ER stress and EMT in renal tubular epithelial cells as well (103).

ER stress and EMP may also regulate one another, according to research findings. Inducing EMT makes cells more vulnerable to ER stress by activating the PERK–eIF2α axis of the UPR. Moreover, PERK–eIF2α signaling is pivotal for maintaining ER homeostasis and is required for EMT cells to disseminate (104). ER stress in colorectal cancer cells is dependent on ZEB-1 induction. Colorectal cancer cells could not mount ER stress in response to microenvironmental stimuli in the absence of ZEB-1 (105).

However, apart from the usual role of ER stress in promoting EMT, some studies have reported the inhibition of EMT by ER stress. Chemical induction of ER stress inhibits EMT and migration in retinal pigment epithelial cells possibly by inhibiting TGF-β signaling (106). Honokiol-induced ER stress markedly inhibits histone deacetylase-3 expression and blocks EMP and metastatic dissemination in gastric cancer (107). Furthermore, melatonin induces ER stress and inhibits EMT *via* calpain-mediated C/EBP-β and NF-κB cleavage in gastric cancer (108). In human glioblastoma cells, sinomenine hydrochloride triggers ER stress, reverses endogenous and exogenous EMT, and inhibits migration and invasion. When ER stress is suppressed, the inhibition of mesenchymal markers (vimentin, Snail, and Slug) is abolished (109). In addition, a recent study shows that metabolism affects the ER stress and modulates EMT. In breast cancer cells, TGF-β-induced EMT could be suppressed by ER stress in response to cholesterol accumulation in the ER (110).

Additionally, ER stress is involved in the MET process. Hyperactivated ER stress is a significant reprogramming barrier that prevents the initial MET step to form induced pluripotent stem cells (iPSCs) from mesenchymal somatic cells (111). Adenosine Deaminase Acting on RNA (ADAR)1-dependent RNA editing could promote MET and induce iPSC reprogramming by alleviating ER stress (112). Overexpression of the ER secretion factor ER protein 29 in breast cancer cells results in enhanced MET phenotypes, including stress fiber loss, E-cadherin upregulation, and vimentin downregulation (113). The relationship between ER stress and EMP remains unknown, particularly in the process of cells shifting along the EMT-MET axis, as evidenced by intermediate hybrid E/M states.

### 3.2 ER stress and chemoresistance

The role of ER stress in promoting or counteracting cancer cell chemoresistance is debatable. There is no agreement on the relationship between ER stress and the development of drug-resistant phenotype in cancer cells. According to some studies, activating ER stress restores chemosensitivity, whereas contradictory results have been reported by other studies. Several investigations have highlighted the role of UPR in the determination of cell fate by either increasing or decreasing the susceptibility of cancer cells to chemotherapy drugs (114). Chemotherapeutic resistance caused by ER stress is common in aggressive tumors. One of the most important mechanisms promoting MDR development is ER stress. In cancer cells, ER stress adaptation results in an MDR phenotype with increased expression of the UPR sensor protein kinase PERK, which mediates Nuclear factor erythroid-derived 2-like 2 (Nrf2)-driven transcription of MDR related protein 1 (MRP1). Silencing PERK signaling inhibits tumor growth and enhances the susceptibility of tumor xenografts to chemotherapy (115). Adaptation to ER stress also improves DNA repair and damage tolerance, thereby increasing the resistance of stressed cancer cells to chemotherapeutics (96). ER stress increases chemoresistance in colon cancer cells by activating eukaryotic initiation factor 2 (eIF2)/ATF4 signaling (116). Moreover, by alleviating ER stress, astragaloside IV sensitizes NSCLC cells to cisplatin (117). Furthermore, ER stress upregulates the expression of the ZNF263–ARHGEF2 pathway, which contributes to ER stress-related treatment resistance (118). When exposed to ER stress, nasopharyngeal carcinoma cells secrete endoplasmic reticulum resident protein 44(ERp44)-containing exosomes, which boost the chemoresistance of neighboring cells (119). Induction of UPR promotes glioma cell metabolism and chemoresistance (120). Moreover, downregulation of ER stress response inhibits autophagy and overcomes temozolomide resistance in melanoma cells (121).

On the contrary, ER stress can counteract cancer cell chemoresistance and mediate cell apoptosis. Tunicamycin, for example, significantly increases chemotherapy-induced apoptosis by inducing ER stress in multidrug-resistant gastric cancer cells (122). Accordingly, our previous study found that tunicamycin-induced ER stress reduces the chemoresistance of hypopharyngeal carcinoma cells both *in vitro* and *in vivo* (**Figure 2B**) **(** 123). WW domain containing oxidoreductase (WWOX) makes epithelial ovarian cancer cells more sensitive to paclitaxel *via* ER stress-induced apoptosis (124). Betulinic acid treatment increases GRP78-dependent ER stress and exerts chemosensitizing effects in breast cancer (125). transmembrane 9 superfamily 4 (TM9SF4) knockdown increases ER stress, reduces cell growth, and induces cell death in chemoresistant breast cancer cells (126). Furthermore, PERK activation induces ER stress and improves the chemosensitivity to taxol treatment in colorectal cancer cells (127), and a combination of 5-FU and withaferin-A upregulates the expression of ER stress sensors and induces PERK axis-mediated apoptosis (128). Hence, a thorough understanding of the opposing roles of ER stress in regulating the drug resistance and sensitivity of cancer cells may have significant implications for the selection of different treatment strategies.

### 3.3 ER stress and CSCs

ER stress plays a crucial role in regulating the functions of stem-like cells. Endodermal differentiation of mouse embryonic stem cells could be induced by the ER stress-inducing agents thapsigargin and tunicamycin (129). ER stress response promotes BMP9-induced bone formation and matrix mineralization in mesenchymal stem cells (130). According to research findings, UPR activates ER stress, which causes rapid loss of stemness in intestinal epithelial cells (IECs) (131). A recent study found that excessive ER stress causes apoptosis in intestinal epithelial stem cells, thereby resulting in aggravated colitis (132). Another study made a similar observation in IECs. ER stress is induced during the transition from stem cells to transit-amplifying cells and mediates stem cell loss in a PERK–eIF2α–dependent manner. Similarly, ER stress disrupts Wnt signaling downstream of nuclear β-catenin, which causes the death of Apc-mutated intestinal epithelial stem cells (133). Furthermore, XBP1 has been shown to decrease the stemness of IECs (134).

In tumors, ER proteostasis is important for maintaining CSC integrity. In breast cancer, stem-like cells express high levels of ER-associated p97, the loss of which activates UPR and alters the expressions of multiple stemness-associated genes, thus leading to the demise of CSCs (135). ER stress also activates UPR, which promotes the differentiation of colon CSCs, thus leading to enhanced chemosensitivity (136). For instance, overexpression of ATF6 and XBP1 reduces the proliferation and stemness of colorectal cancer cells by activating PERK signaling (137). Moreover, brefeldin A, an inducer of ER stress in eukaryotic cells, inhibits CSC-like properties in colorectal (138) and breast cancer cells (139). Interestingly, CSCs are sensitive to the mitochondrial targeting antibiotic doxycycline, which induces ATF4-mediated ER stress and leads to apoptosis selectively in the cancer stem-like cells (140). Furthermore, the adaptation to ER stress drives the malignancy and drug resistance of tumor cells. Study of the relationship between CSCs and adaptation to ER stress has revealed that the proportion of apoptosis-resistant CSCs is elevated in ER stress-resistant melanoma. Similarly, Homeobox B9 (HOXB9) regulates the self-renewal of CSCs and antagonizes ER stress-induced apoptosis by modulating the miR–765–FOXA2 axis in melanoma cells (141). Therefore, apart from the role of ER stress in CSC differentiation and apoptosis, mechanisms involving ER stress regulation on CSC plasticity, such as quiescence and activation, and the interchange of coexistence of diverse phenotypes need to be further investigated.

### 3.4 ER stress and angiogenesis and VM

Evidence suggests that ER stress alters the expression and activity of vascular growth factors, thereby modulating the functions of vascular endothelial cells and tumor angiogenesis (142–145). Hepatitis B virus small envelope protein-induced ER stress activates UPR signaling, thereby increasing the expression and secretion of vascular endothelial growth factor (VEGF) A and, consequently, the angiogenic capacity of hepatocellular carcinoma cells (146). In malignant glioma, IRE1 is a critical regulator of tumor angiogenesis and metastasis. Inhibiting IRE1α is associated with a decrease in proangiogenic cytokines, such as VEGFA, IL-1beta, IL-6, and IL-8, and an increase in antiangiogenic gene transcripts (147). In collaboration with hypoxia-inducing factor 1α (HIF1α), XBP1s drive the angiogenesis and progression of TNBC as the downstream transcription factor of IRE1α (148). However, ER stress has been shown to drive antiangiogenic responses. ER stress-induced miR-153 expression in breast cancer cells activates IRE1α and XBP1, which inhibits HIF1α expression and tumor angiogenesis by decreasing VEGFA production (149). Neuronal ER stress inhibits myeloid cell-induced vascular regeneration by promoting the degradation of IRE1α-dependent netrin-1 (150). Moreover, C/EBP homologous protein-10 (CHOP-10) can activate an antiangiogenic response in postnatal neovascularization under ER stress (151). By activating p38-mediated ER stress, low-intensity pulsed ultrasound increases apoptosis and inhibits angiogenesis in endothelial cells (152).

The majority of studies linking ER stress to tumor angiogenesis have focused on VEGF and other factors with vasomodulatory properties in angiogenic cascades modulated by the UPR. However, the influences of ER stress on the VM phenotypes of cancer cells remain poorly understood. TNBC cells with mesenchymal phenotypes form tubular VM networks in three-dimensional (3D) matrigel cultures. For the first time, Liu et al. reported that the activation of ER stress reduces VM phenotypes in TNBC cells *via* regulation of TGF-β1/Smads and β-catenin signaling pathways (**Figure 2B**) **(** 153). As a potential transdifferentiation event indicative of the unique capability of certain aggressive tumor cells associated with EMT and stemness, VM plasticity has profound implications in tumor progression. Therefore, the potential regulatory mechanisms involved in ER stress and VM should be clarified to facilitate the development of targeted therapies that prevent tumor angiogenesis and consequently impede tumor progression.

### 3.5 ER stress, calcium signaling, and EMP

The ER must maintain a tightly controlled oxidizing and Ca^2+^-rich folding environment for protein synthesis, folding, and modification. ER-resident chaperones, such as immunoglobulin binding protein, calreticulin, calnexin, and protein disulfide isomerases, play critical roles in ER protein folding and Ca^2+^ buffering. ER-Ca^2+^ depletion may disrupt ER homeostasis and the balance between protein folding load and capacity (154). Many aspects of tumor activity, including proliferation, angiogenesis, invasion, EMT, and drug resistance, involve calcium signaling pathways (155). TGF-β-induced EMT is associated with alterations in ER calcium homeostasis in human breast cancer cells (156). Calreticulin is an ER-resident multifunctional protein that promotes TGF-β-induced EMT (**Figure 2B**) **(** 157). Moreover, the effect of cyclophilin B (CypB) regulation on Slug expression in renal tubular epithelial cells is dependent on its interaction with calreticulin and calreticulin-dependent calcium signaling in the ER lumen (158). Another study found that the ER transmembrane protein transmembrane and coiled-coil domains 1 (TMCO1) is important for maintaining calcium homeostasis, promoting EMT in human gliomas, and inducing cell migration and invasion (159). Furthermore, EMT remodels Ca^2+^ influx in breast cancer cells, possibly by changing the functions of the store-operated Ca2+ channel poreforming subunit ORAI1 and transient receptor potential canonical type 1 (TRPC1) channels (160). In terms of the regulation of specific mesenchymal markers, ER ATPase inhibitor thapsigargin is an inducer of vimentin in breast cancer cells, which involves store-operated Ca^2+^ entry (161). Calreticulin induces EMT in pancreatic cancer *via* intracellular free Ca^2+^-dependent, thapsigargin-induced acute stress and IRE1α-mediated chronic ER stress (**Figure 2B**) **(** 162).

EMP-associated hybrid E/M or pEMT states are distinct from classical EMT and could confer unique malignant properties to tumor cells (17, 22, 163). Recent studies have revealed the links between calcium signaling and pEMT (**Figure 2B**). Prolonged calcium signaling induces pEMT in carcinoma cells, which is accompanied by the internalization of membrane-associated E-cadherin and other epithelial proteins and an increase in cellular migration and invasion (164). Tumor cells within a mesenchymal state have more intracellular calcium, and ER, as one of the Ca^2+^ stores in cells, might play a role to facilitate intracellular Ca^2+^ reaching levels sufficient for P-EMT. However, the point of convergence of the P-EMT and complete EMT transitions remains unclear. It was reported that ER stressor thapsigargin increased cytosolic Ca^2+^ concentration, while it was also shown to increase levels of active TGF-β1 (165). It is possible that Ca^2+^ dysregulation induced the mesenchymal transformation of cells and then TGF-β1 activation leads to a complete EMT response. Thus, extracellular signals prompt an increase in Ca^2+^ flux, and the release of Ca^2+^ from ER stores mediates EMT in multiple ways. Therefore, exploring the potential therapeutic benefits of targeting Ca^2+^ signaling to block EMP in cancer cells could provide a novel complement to standard therapies.

## 4 Discussion

Cellular plasticity has emerged as a well-recognized mode of therapeutic resistance in various cancers in recent years. Tumor progression, metastasis, and drug resistance are driven by cellular plasticity. The mechanisms governing this cell state switch have also been elucidated. Persistent ER stress, an emerging cancer hallmark, is caused by various factors that disrupt ER homeostasis in malignant cells. It is well known that unresolved ER stress promotes tumor cell malignancy and drug resistance, thus contributing to the acquisition of EMP, stemness, and drug resistance plasticity to promote tumor progression.

However, our understanding of the interactions between ER stress and cancer cell plasticity and the levels and specificities of regulation for a specific type of plasticity is still limited and warrants further investigation. Decoding how the ER stress pathway regulates cell plasticity is a major challenge for researchers and necessitates defining the rationale for drug design and application. Deciphering the molecular connections between ER stress and cancer cell plasticity will definitely contribute to the development of new therapeutic strategies that, when combined with existing anticancer treatments, will provide better clinical responses in patients.

## Author contributions

HW prepared figures and wrote the first draft of the manuscript. KM conceptualized and edited the manuscript. All authors contributed to the article and approved the submitted version.
